# METTL3-Mediated m^6^A RNA Modification Regulates Corneal Injury Repair

**DOI:** 10.1155/2021/5512153

**Published:** 2021-10-22

**Authors:** Yarong Dai, Maosheng Cheng, Siyan Zhang, Rongsong Ling, Jieqi Wen, Yifan Cheng, Boxuan Huang, Jinrong Li, Caifeng Dai, Shiqing Mao, Shuibin Lin, Huangxuan Shen, Yizhou Jiang

**Affiliations:** ^1^Institute for Advanced Study, Shenzhen University, 518067, China; ^2^Anhui Medical University, 230032, China; ^3^Center for Translational Medicine, Precision Medicine Institute, The First Affiliated Hospital, Sun Yat-sen University, 510064, China; ^4^State Key Laboratory of Ophthalmology, Zhongshan Ophthalmic Center, Sun Yat-sen University, 510064, China; ^5^Center for Reproductive Medicine, Department of Obstetrics and Gynecology, Qilu Hospital of Shandong University, 250012, China

## Abstract

Limbal stem cells are essential for continuous corneal regeneration and injury repair. METTL3-catalyzed N6-methyladenosine (m^6^A) mRNA modifications are involved in many biological processes and play a specific role in stem cell regeneration, while the role of m^6^A modifications in corneal injury repair remains unknown. In this study, we generated a limbal stem cell-specific METTL3 knockout mouse model and studied the role of m^6^A in repairing corneal injury caused by alkali burn. The results showed that METTL3 knockout in the limbal stem cells promotes the *in vivo* cell proliferation and migration, leading to the fast repair of corneal injury. In addition, m^6^A modification profiling identified stem cell regulatory factors AHNAK and DDIT4 as m^6^A targets. Our study reveals the essential functions of m^6^A RNA modification in regulating injury repair and provides novel insights for clinical therapy of corneal diseases.

## 1. Introduction

The integrity and function of the cornea depend on the self-renewing properties of the corneal epithelium. The corneal limbus locates at the junction of the cornea, the conjunctiva, and the sclera. Normal corneal epithelial cells and conjunctival epithelial goblet cells are derived from limbal stem cells [1]. Misregulation of limbal stem cells often results in visual defects, and regulation of limbal stem cell regeneration is a promising strategy for treating eye diseases [2, 3]. Limbal stem cells maintain the corneal physiology and biochemistry and regulate its nutrition, immune response, and integrity. Keratin 14 (K14) is a marker of limbal stem cells, and K14^CreER^ was observed migrating centripetally from the limbus to the central cornea. Stem cell fate decisions depend on the interaction of extracellular signals from the niche with intracellular signaling cascades and transcriptional programs [4]. Therefore, studying proliferation, differentiation, and the regulatory mechanism of limbal stem cells can provide new methods and insights into treating corneal defects in various ocular diseases.

METTL3 (methyltransferase like 3, also known as MTA70) was originally identified as a methyltransferase responsible for m^6^A modifications of mRNAs [5]. METTL3-mediated m^6^A modifications regulate multiple mRNA processing events, including splicing, export, translation, and stability [6]. Physiologically, m^6^A misregulation is closely related to developmental diseases and DNA lesion repair [7–9]. Studies from our group and others have revealed that m^6^A modifications impact stem cell fate determination in embryonic stem cells and different tissue progenitor cells via NANOG, SOX2, NR5A2, and MYC [7–9]. Besides, the knockdown of METTL3 dramatically delays the ultraviolet-responsive DNA damage repair [10]. Despite this growing appreciation of m^6^A mRNA modification pathways' biological significance, the functions and mechanisms for how m^6^A might regulate limbal stem cells and corneal injury repair remain poorly understood [11–13].

In this study, we established a limbal stem cell-specific METTL3 knockout mouse model. We investigated the role of METTL3-mediated m^6^A modification in the regulation of corneal injury repair using an alkali burn model. Our study identified METTL3 as a critical factor for corneal injury repair and suggested that manipulation of METTL3 and m^6^A modifications could be a promising therapeutic strategy for treating corneal diseases.

## 2. Materials and Methods

### 2.1. Materials

Rabbit anti-*METTL3* (Abcam, ab195352) and mouse anti-cytokeratin 14 (Abcam, ab7800) antibodies were used. Mouse anti-Ki67 (Therm), rabbit anti-cytokeratin 10 (Abcam, ab76318), anti-cytokeratin 5 (ab53121, Abcam), anti-cytokeratin 1 (ab93652, Abcam), and anti-CD31 (bs-20322R, Bioss) antibodies were also used. A PAS Staining kit (G1008-100, Xiangbo), an H&E Staining kit (G1120-100, Solarbio), an immunohistochemical kit (SA102, Boster), an OCT frozen section embedding agent (4583, OCT), an AHNAK antibody (sc-390743, a Santa Cruz Biotechnology), a REDD1 antibody (10638-1-AP, Proteintech), and a GAPDH antibody (60004-1-Ig, Proteintech) were used in this study.

### 2.2. METTL3 Knockout Mouse Model

Mettl3^fl/wt^ mice were generated as previously described. Mettl3^fl/wt^ mice were crossed with K14^CreER^ mice to obtain K14^creER^/Mettl3^fl/fl^ (cKO) mice. Mettl3 cKO and control mice were injected with tamoxifen and then were subjected to corneal alkali burn treatment. The right eye was the experimental eye, and the left eye was the control eye. The mice were sacrificed at 24 hours, 7 days, 14 days, 35 days, and 56 days after injury. Six mice were taken from each period. Both eyes were removed, frozen in OCT (*n* = 4), fixed in 4% paraformaldehyde, and embedded in conventional paraffin (*n* = 2).

### 2.3. Alkali Burn Model

Tribromoethyl alcohol 12-14 *μ*l/g was administered via intraperitoneal injection for general anesthesia. For local anesthesia, Erkain eye drops were added to the right eye. After 10 minutes, the excess fluid was scrubbed from the conjunctival sac with a swab; the left eye was treated with methylcellulose to maintain ocular surface moisture. After the mice were fully anesthetized, they were placed in a supine position on a micromanipulation platform. Circular filter paper with a diameter of 2 mm was immersed in 1 mol/L sodium hydroxide solution for 10 s and was removed with ophthalmic micro tweezers. The excess liquid was wiped off with dry gauze, and then, the filter paper was placed in the center of the mice's cornea for 15 s. After removing the filter paper, the eye was immediately rinsed with normal saline for 10 min, and erythromycin eye ointment was applied to the mice's right eye.

### 2.4. Corneal Fluorescein Staining Assessment

One drop of normal saline was used to wet fluorescein sodium test paper, and 10 *μ*l of fluorescein sodium was dropped into the center of the cornea of mice to cause the fluorescein sodium to enter the corneal injury site. The mice were made to blink three times to fill their eyes with sodium fluorescein. Excess sodium fluorescein staining solution was wiped away with a cotton swab at the conjunctival sac. Cobalt blue light was used under a microscope to observe and photograph the corneas. The cornea was evenly divided into 4 quadrants. After each quadrant was scored separately, the quadrants' values were added to obtain the corneal fluorescein sodium staining score. The scoring methods in each quadrant were as follows: no staining was 0 points; a few stained dots but fewer than 30 dots were assigned 1 point; the number of dots in the punctate staining was more than 30, but no flake or block staining was assigned 2 points; diffuse flake or block staining was assigned 3 points.

### 2.5. Hematoxylin-Eosin Staining

The tissues of mice were fixed with 4% paraformaldehyde (Shenggong, Shanghai, China), washed, dehydrated, transparentized, immersed in wax, and cut into sections. The dried sections were immersed in a dyeing vessel containing xylene I and xylene II for dewaxing. Sections were sequentially immersed in different alcohol concentrations, double-distilled water (for hydration), stained in HE, dehydrated, and sealed. The prepared tissue sections were observed under an optical microscope and photographed using a microimaging system.

### 2.6. Immunofluorescence Staining

The sections were dewaxed, followed by the antigen repair method, and then washed three times with PBS. The sections were blocked with 5% normal goat serum for 1 h at room temperature and then were incubated with a primary antibody at 4°C overnight. After washing with PBS, sections were incubated with an appropriate secondary antibody for 1 h at room temperature. Sections were stained with DAPI (Beyotime, Haimen, Jiangsu, China) for 15 min and then were observed under a fluorescence microscope (Olympus/BX51) after three 5 min washes with PBS. For double immunofluorescence, sections were incubated consecutively with pairs of primary and secondary antibodies. Sections were examined under a fluorescence microscope (Olympus/BX51, Tokyo, Japan). ImageJ was used to evaluate the fluorescence intensity of Ki67, K14, K5, K10, K1, and CD31.

### 2.7. PAS Staining of Conjunctival Goblet Cells

Periodic acid colorless magenta staining, referred to as the PAS staining method, is one of the staining methods commonly used to detect neutral mucus in paraffin section technology. The density and distribution of conjunctival goblet cells can be observed by PAS staining (PAS staining kit).

### 2.8. RNA-seq

Total RNA was extracted with TRIzol and was assessed with a NanoPhotometer and Agilent 2100 BioAnalyzer. Sequencing libraries were prepared using Illumina's NEBNext, UltraTM RNA Library Prep Kit. After the library was built, a Qubit2.0 fluorometer was used for preliminary quantification, which was performed on libraries that were diluted to 1.5 ng/*μ*l. An Agilent 2100 BioAnalyzer was used to test the library's insert size, and Illumina sequencing was performed after the library was validated. The reference genome and gene model annotations were downloaded directly from the Genome website. HISAT2 V2.0.5 was used to construct the reference genome index, and HISAT2 V2.0.5 was used to compare paired-end clean reads with the reference genome.

### 2.9. MeRIP-seq

Total RNA (500 ng-20 *μ*g) was extracted with TRIzol. The methylation modification spectra of mRNA, lncRNA, and circrRNA can be detected simultaneously using the latest rRNA removal technology. FastQC was used to analyze sequencing data quality to obtain information such as sequencing quality distribution, base content distribution, and repeat sequencing fragment ratio. This study used the HISAT2 software to compare the filtered effective sequencing data with the HG38 reference genome, and then, the comparison rate was calculated. An important step in analyzing m^6^A levels through peak calling was standard quality testing of the data using exome peak. Based on identified m^6^A peaks, a metagene plot diagram was mapped. The HOMER software was used to perform motif analysis of peaks. The genes with m^6^A modifications obtained by the analysis were annotated with gene ontology (GO), based on biological process (BP), molecular function (MF), and cellular component (CC) in the GO database. Fisher's test calculated the significance level (*P* value) of each GO term to screen out significant terms. Pathway annotation was carried out on select m^6^A genes based on the KEGG database. Fisher's test calculated the significance level (*P* value) to screen out the significant pathway terms enriched for genes with m^6^A modifications.

### 2.10. RT-qPCR

Total RNA was extracted with TRIzol. cDNA was synthesized from 1 *μ*g total RNA by Roche 11483188001 kit. Quantitative-PCR (qPCR) was carried out in a KiCqStart SYBR Green qPCR ReadyMix with 2 *μ*l of first-strand cDNA and 500 nM primers in a final volume of 20 *μ*l. Reactions were begun with an initial denaturation at 95°C for 10 min. 45 cycles were run: 95°C for 10 s, 60°C for 30 s, and 72°C for 15 s. A melting curve was run to detect the desired amplicon. Amplicon size was verified by ECO Real-time PCR machine (Illumina). The housekeeping gene *β*-actin was used for normalization. qPCR primers are METTL3 (F: TTGTCTCCAACCTTCCGTAGT and R: CCAGATCAGAGAGGTGGTGTAG), DDIT4 (F: TGAGGATGAACACTTGTGTGC and R: CCAACTGGCTAGGCATCAGC), AHNAK (F: TACCCTTCCTAAGGCTGACATT and R: TTGGACCCTTGAGTTTTGCAT), and *β*-actin (F: CATGTACGTTGCTATCCAGGC and R: CTCCTTAATGTCACGCACGAT).

### 2.11. Western Blot

The proteins were resolved by 12% SDS-PAGE and transferred onto the PVDF membrane (Immobilon). After primary antibody (1 : 2000) incubation at 4°C overnight, the secondary AHNAK (sc-390743, Santa Cruz Biotechnology), REDD1 (10638-1-AP, Proteintech), or GAPDH (60004-1-Ig, Proteintech) antibodies (1 : 2000) and ECL detection kit (Immobilon) were used to visualize target proteins.

### 2.12. Statistical Analysis

Data are expressed as the mean ± standard deviation (SD) or standard error (SE). All statistical analyses were performed using Excel or GraphPad Software. Significance was set as ^∗^*P* < 0.05, ^∗∗^*P* < 0.01, ^∗∗∗^*P* < 0.001, and ^∗∗∗∗^*P* < 0.0001 as assessed by two-tailed Student's *t*-tests or one-way analysis of variance.

## 3. Results

### 3.1. METTL3 Knockout Promotes Corneal Injury Repair

**Figure 1:**
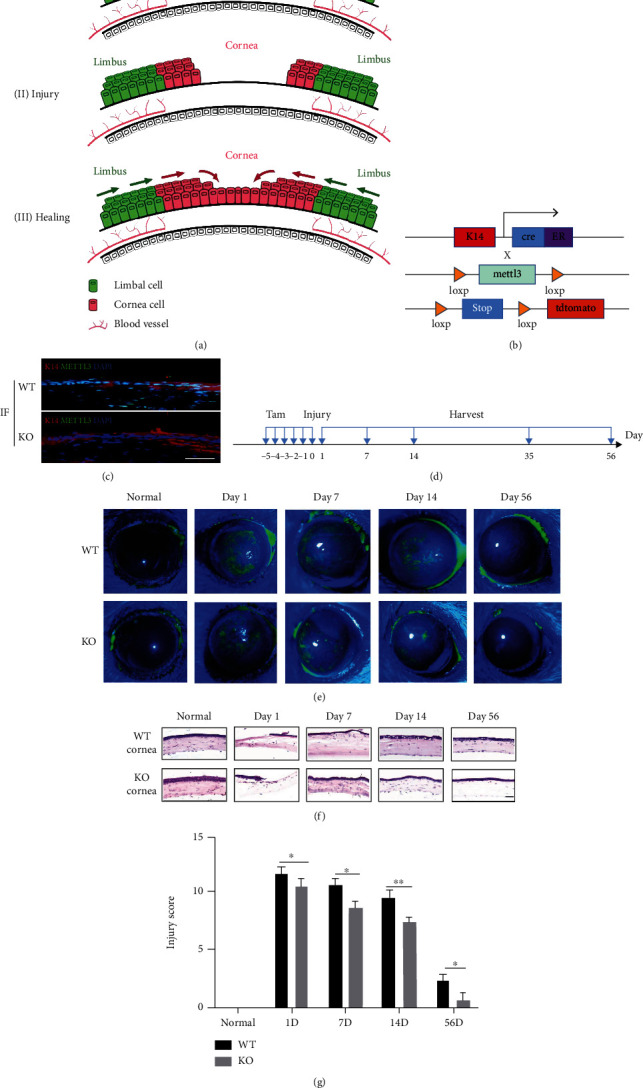
K14^creER^/Mettl3^fl/fl^ mice and cornea damage model construction. (a) Corneal and limbus homeostasis, damage, and repair process. (b) K14^creER^/tdTomato^+/+^/Mettl3^fl/fl^ mouse crossing strategy. (c) Immunofluorescence of K14^creER^/Mettl3^wt/wt^ and K14^creER^/Mettl3^fl/fl^ mouse limbal cells (scale: 100 *μ*m). (d) Two- to three-month-old mice were induced with tamoxifen injection, eyes were subjected to alkali-induced injury by five days of continuous induction, and samples were collected at several time points after injury. (e–g) Corneal epithelium in 2- to 3-month-old mice with alkali burns. (e) Degree of corneal injury with sodium fluorescein staining at different time points. (f) H&E staining of frozen sections of corneal injury at different time points (scale: 100 *μ*m). (g) The corneal injury score was obtained by fluorescein sodium staining.

We first generated an alkali burn model using limbal stem cell-specific METTL3 conditional knockout mice (Figures 1(a) and 1(b)) to study the role of METTL3-mediated m^6^A modifications in the regulation of limbal stem cell function and corneal injury repair. The deletion of METTL3 in keratin 14-positive cells was confirmed by keratin 14 (K14) and METTL3 immunofluorescence staining (Figures 1(c) and 1(d)). Then, the alkali burn model was established, and Mettl3 cKO mice and wild-type (WT) mice were treated with sodium fluorescein at different times. Through sodium fluorescein staining of mouse corneal cell layers, we found that one day after the injury, eyes in the WT mice showed a wide range of diffuse patch staining, indicating the defective function to exclude dye penetration due to corneal injury. The staining intensity decreased on day 7 and day 14, but still obvious sodium fluorescein staining could be detected in the eyes. On day 56, little sodium fluorescein staining could be found, suggesting that the corneal function successfully recovered to exclude sodium fluorescein. Interestingly, METTL3 knockout in the corneal stem cells results in quicker restoration of eye function to exclude sodium fluorescein, suggesting that METTL3 deletion promotes the corneal injury repair in the alkali burn model (Figure 1(e)).

At different time points after injury, significant differences in phenotype between wild-type mice and Mettl3 cKO mice were observed (Figure 1(f)). On day 1, the corneal epithelial cell layer and the anterior elastic layer of the WT and cKO mice were all destroyed by alkali burn, and the corneal stroma layer was loosely arranged. Seven days after the injury, the corneal epithelial layer of WT mice was almost restored; the corneal basal layer was still loosely arranged; most of the neovessels were orderly and dense; the blood vessels were flourishing. The corneal epithelium of the Mettl3 cKO group of mice became complete, the stromal layer was tightly packed, the neovascularization was sparse, and the number of vessels was small. On the fourteenth day, the corneal epithelial cell layer of the WT mice was complete, and the stromal layer had less neovascularization. In contrast, the corneal epithelial cell layer of Mettl3^−/−^ group mice was relatively complete, and no neovascularization was observed in the stromal layer. After fifty-six days, the corneal epithelial cell layers of both the WT and Mettl3^−/−^ mice were intact, and there was no neovascularization in the stromal layer (Figure 1(f)). Statistical analysis showed that the damage repair rate in Mettl3 cKO mice was slightly faster than in WT mice (Figure 1(g)). Overall, our data revealed that METTL3 knockout in the corneal stem cells promotes the corneal injury repair.

### 3.2. Effect of METTL3 on the Repair of Corneal Epithelial Injury

**Figure 2:**
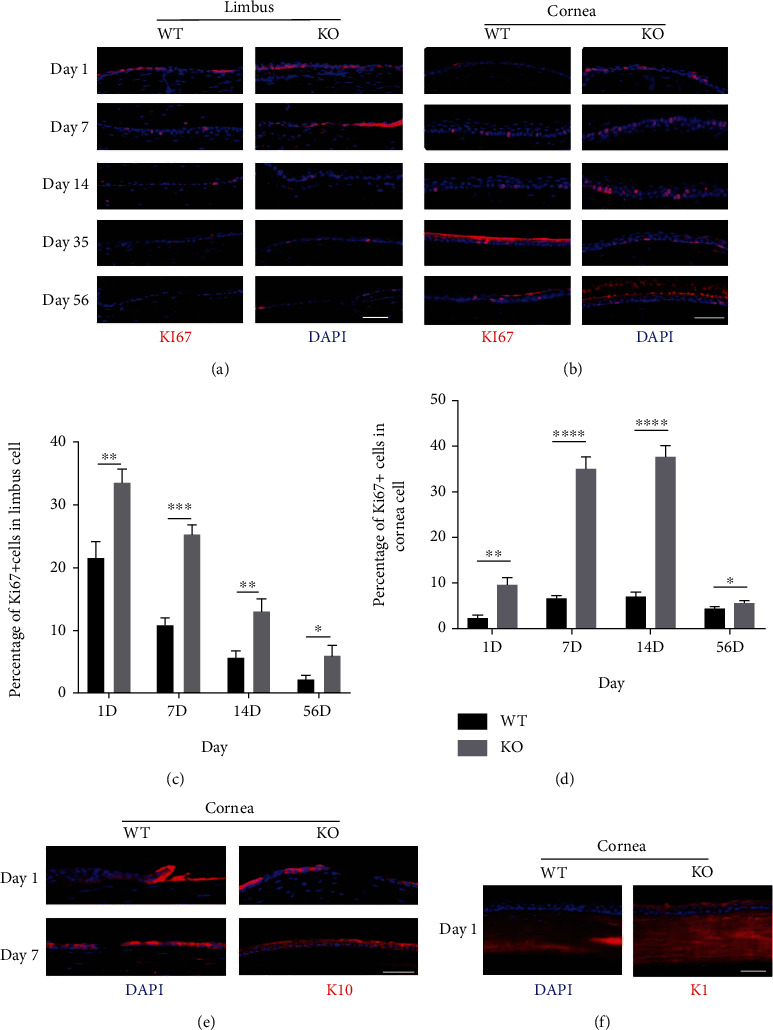
Effects of METTL3 knockout on proliferation in corneal injury repair. (a) Ki67 staining of frozen sections of limbus at different time points (scale: 200 *μ*m). (b) Ki67 staining of frozen sections of injured corneas at different time points (scale: 200 *μ*m). (c, d) Percentage of Ki67^+^ cells in the (c) limbus and (d) cornea. (e–f) K10 and K1 staining of frozen sections of injured corneas (scale: 200 *μ*m).

We further performed lineage tracing and immunohistochemical analysis of keratin Ki67, K14, K10, K1, and CD31 to investigate the role of METTL3 in corneal injury repair. Our results showed that the expression of Ki67 in the limbal epithelium and corneal epithelium of WT mice was lower compared to the Mettl3 cKO mice (Figures 2(a)–2(d)). There was a positive correlation between the expression of K1 and K10. The lower Ki67 expression and the correlation between K1 and K10 expression suggested that the increased expression of keratin K1/K10 in corneal injury was one of the reasons for the accelerated proliferation of the corneal epithelium. Moreover, the expression levels of K1/K10 in the corneal epithelium of WT mice were lower than they were in the Mettl3 cKO mice (Figures 2(e) and 2(f)).

**Figure 3:**
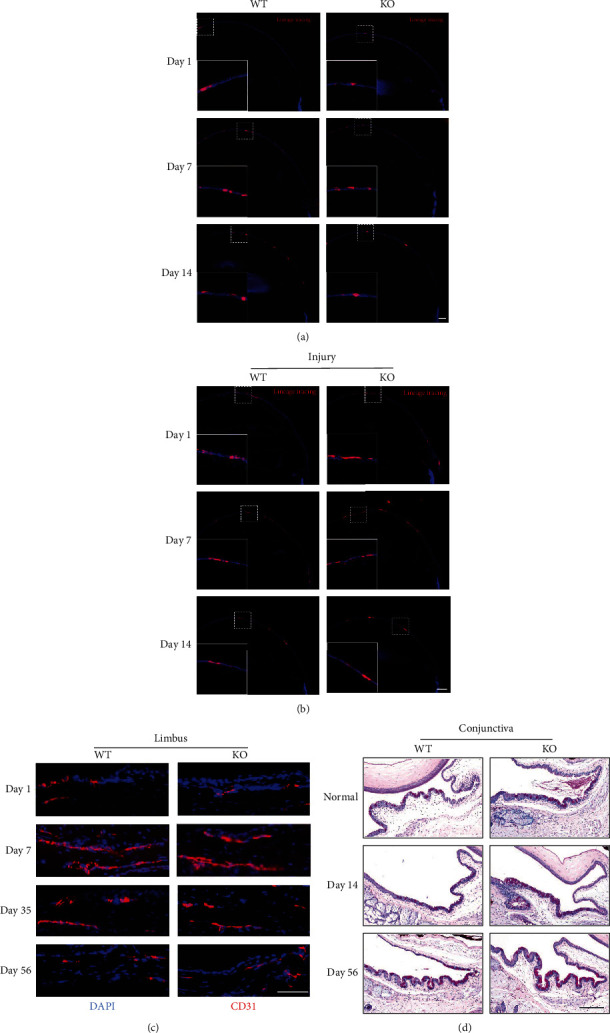
Effects of METTL3 knockout on migration, goblet cells, and neovascularization in corneal injury repair. (a) Representative images of Tomato^+^ cell (red) derived from K14^+^ cells after injecting tamoxifen (TAM) under normal conditions (scale: 50 *μ*m). (b) Representative images of Tomato^+^ cell (red) derived from K14^+^ cells after injecting tamoxifen (TAM) under injury conditions (scale: 50 *μ*m). (c) CD31 staining of frozen sections of injured corneas at different time points showed neovascularization (scale: 200 *μ*m). (d) PAS staining of the conjunctiva of WT mouse and Mettl3 cKO mouse (scale: 200 *μ*m).

*In vitro* lineage tracing showed that the expression level of tdTomato^+^ cells derived from K14^+^ cells of WT mice and Mettl3 cKO mice showed no significant difference under normal physiological conditions. Under injury conditions, compared with WT mice, the expression level of tdTomato^+^ cells derived from K14^+^ cells of Mettl3 cKO mice was significantly increased (Figures 3(a) and 3(b)). To explore the effect of METTL3 on corneal repair after injury, we applied CD31 immunofluorescence to assess the vascular area at the injured site on the corneal limbus. The vascular area of WT mice was larger than that of Mettl3 cKO mice (Figure 3(c)). Moreover, the number of goblet cells in WT mice was less than that in the Mettl3 cKO group (Figure 3(d)). All the above results suggest that METTL3 depletion accelerated the speed of the corneal epithelial repair after injury.

### 3.3. METTL3 Targeting AHNAK and DDIT4 in the Corneal Limbus

**Figure 4:**
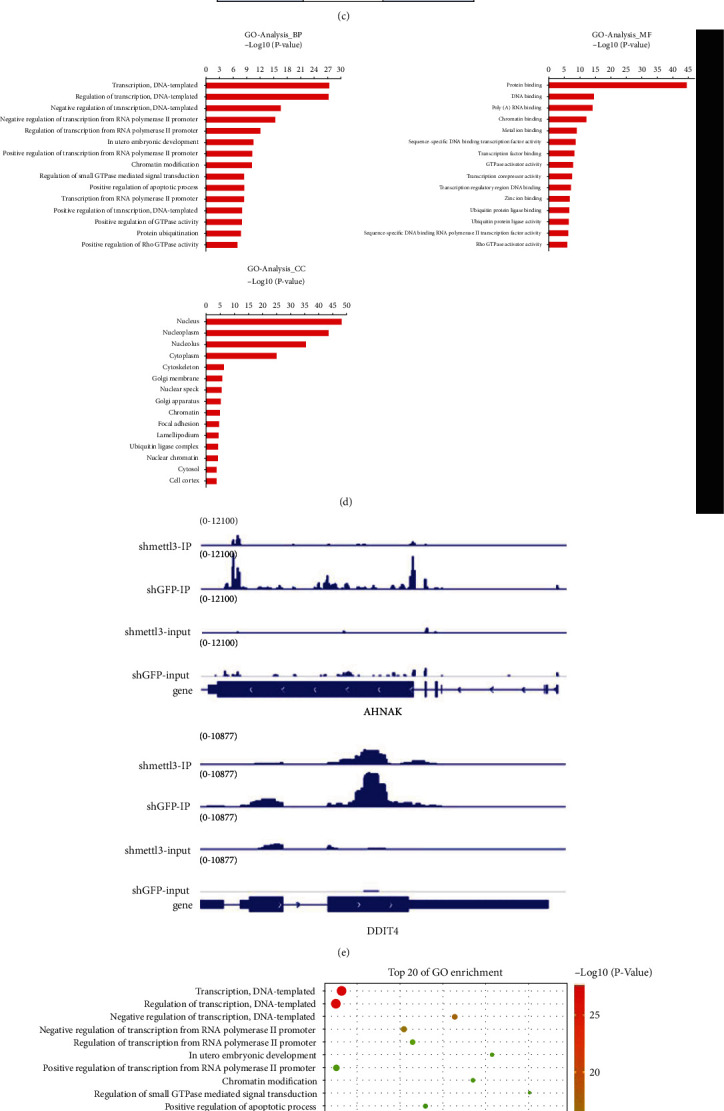
Methylated RNA immunoprecipitation sequencing (MeRIP-seq) and RNA sequencing (RNA-seq) analysis of HCES3 cells with METTL3 knockdown. (a) Volcano map of differentially expressed genes. Red spot, genes that were significantly upregulated; green spot, genes that were significantly downregulated; and blue spot, genes that were not significantly downregulated. (b) Metagene profiles of m^6^A distribution across the transcriptome of HCES3 cells with METTL3 knockout. (c) Consensus motifs enriched with m^6^A peaks from transcripts identified in HCES3 cells with METTL3 knockout (2224 peaks). (d) GO analysis results of genes with m^6^A modifications. (e) The abundance of m^6^A in AHNAK and DDIT4 transcripts in METTL3 knockdown HCES3 cells was detected and mapped using Integrative Genomics Viewer (IGV). (f) GO mapping of genes with m^6^A modifications. (g) Western blot of gene expression in HCES3 cells after treatment with shMETTL3. (h) Gene expression (relative to GAPDH) quantified by RT-qPCR.

To identify potential mRNAs targeted by METTL3 for the induction of m^6^A methylation in corneal epithelial repair, RNA sequencing and m^6^A sequencing were performed in METTL3 knockdown HCES3 cells. A volcano map of differentially expressed genes was generated (Figure 4(a)), representing multiple gene expression changes in different samples. After METTL3 knockdown, 59 genes were significantly downregulated, and 47 genes were significantly upregulated (Figure 4(a)). We next performed m^6^A profiling to identify the m^6^A targets in HCES3 cells. Metagene analysis showed that m^6^A was mainly located near the coding sequence (CDS) region (Figure 4(b)). Sequence motifs were identified from the m^6^A peaks, and they were found to be consistently enriched in specific motif GGAC (Figure 4(c)). The genes with differential methylation were analyzed by GO. For the genes with m^6^A modifications, GO analysis was conducted to assess the samples from BP, MF, and CC perspectives. The first 15 significantly enriched methylated genes of each enrichment group are listed in Figure 4(d), and the significantly enriched methylated genes in the first 20 GO analyses are displayed. We also found that the enriched genes involved cell proliferation and migration (Figure 4(f)). Through MeRIP-seq analysis, we found that AHNAK and DDIT4 appeared to exhibit a decrease in the m^6^A-modified peak after METTL3 knockdown. It was speculated that AHNAK and DDIT4 were downstream target genes of METTL3. Next, we used the Integrative Genomics Viewer (IGV) to detect and map the m^6^A abundance of AHNAK and DDIT4 transcripts in the METTL3 knockdown HCES3 cells (Figure 4(e)). Furthermore, the western bolt and RT-qPCR of HCES3 cells treated with shMETTL3 showed that the expression of AHNAK and DDIT4 was downregulated obviously (Figures 4(g) and 4(h)). In summary, these data suggested that AHNAK and DDIT4 are potential m^6^A targets of METTL3.

## 4. Discussion

Very little was found in the literature on the question of METTL3 function in corneal injury repair. The corneal epithelium loses its regenerative ability and stability after injury, leading to corneal opacity, which impairs visual function. Our study found that knocking out METTL3 facilitates limbal stem cell proliferation and migration. Besides, Mettl3 KO mice present faster corneal injury repair compare to the wild-type mice. These results corroborate the findings of a great deal of the previous work in knocking down METTL3 promote cell growth and self-renewal [14].

The previous study showed that limbal stem cells significantly inhibit vascular endothelial cells' formation, and loss of limbal stem cells will lead to a large amount of neovascularization [15, 16]. Our result showed that knocking out of METTL3 facilitates the neovascularization. Limbal stem cell transplantation is a promising strategy for repairing the defect of limbal tissue and restoring the number of stem cells; thus, inhibiting the corneal epithelium's conjunctiva and neovascularization maintains the cornea's transparency and achieves reconstruction of the ocular surface. Further understanding of the molecular mechanisms regulating limbal stem cell function is necessary to develop a better solution for corneal injury.

The m^6^A methylation of mRNA is an epigenetic modification involved in the fate of eukaryotic stem cells. In normal hematopoiesis, m^6^A controls the differentiation of hematopoietic stem cells without affecting their self-renewal or proliferation. Loss of METTL3 leads to an increase in symmetrical self-renewing cell division [17]. ACTS is an amplifier that selectively promotes the expression of already dominant genes in embryonic stem cells when METTL3 is knocked out. As a result, epithelial stem cells undergo accelerated differentiation and cell death [18]; in this study, our results, which were based on a constructed alkali burn model using keratin 14 (K14)^CreER+^/Mettl3^fl/fl^ mice, showed that Mettl3 cKO might increase the proliferation and differentiation of limbal stem cells.

There is growing evidence to support the idea that the corneal epithelium is regenerated from stem cells located in limbic niches around the cornea. Limbal epithelial stem cell cultures have long been used to prepare grafts that can restore human corneal epithelial defects; thus, exploring the biological functions of limbal stem cells may lay a foundation for improving limbal stem cell therapy [19–21]. Corneal limbus stem cells express Ki67, which plays a vital role in maintaining cell proliferation. However, K14 is mainly expressed in basal epithelial cells of human and mouse corneal limbus and basal epithelial cells in the center of the cornea, and it is a marker of basal epithelial cells with mitotic activity and participates in cell proliferation and migration processes. The expression and fate of K14 could be traced by the expression of tdTomato in cells. This result indicated that limbal stem cells expressing K14 migrated from the limbus to the corneal epithelium to proliferate, differentiate, and repair. K10 and its ligand K1, often used as indicators of normal terminal differentiation, were mainly present in the basal layer and the layers above the basal layer. K10 and K1 were not expressed in normal limbal and corneal epithelium, and there was a positive correlation between their expression. This finding suggested that the increased expression of keratin K1 and K10 in corneal injury was one of the reasons for the accelerated proliferation of the corneal epithelium. Immunofluorescence data for Ki67, K14, K10, and K1 showed that the proliferation, differentiation, and migration rate of the damaged cells were accelerated in Mettl3 cKO mice, while these rates were much lower in the WT mice. These findings might indicate that METTL3 knockout promotes the proliferation, self-renewal, differentiation, and migration rate of stem cells to some extent, thus accelerating the damage repair process.

AHNAK and DDIT4 are involved in stem cell maintenance, proliferation, and differentiation [22–25]. A previous study reported that overexpression of AHNAK inhibited glioma cell proliferation, invasion, Ki67 expression, and induced apoptosis [26]. Lim et al. found that AHNAK inhibition could upregulate endogenous c-Myc, promoting the generation of induced pluripotent stem cells [22]. Like our results, Ki67 expression increased after m^6^A modification of AHNAK, indicating that cell proliferation was increased. DDIT4 is directly related to the differentiation potential of mesenchymal stem cells [27]. The gain of function and loss of function analysis showed that DDIT4 activity was directly related to the regulation of the mTOR signaling pathway and the expression and differentiation of pluripotency genes [27]. In conclusion, METTL3 may affect the growth and metabolism of limbal stem cells by regulating the expression of AHNAK and DDIT4, which plays a vital role in cellular regeneration and enhances the function of m^6^A in the process of corneal repair after injury.

## 5. Conclusions

Using *in vivo* limbal stem cell-specific knockout model, we uncovered the essential role of METTL3-mediated m^6^A modification in the regulation of limbal stem cell function and corneal injury repair, suggesting that targeting the METTL3 and m^6^A work's modification pathway could be a strategy for the therapy of corneal diseases.

## Data Availability

The experiment data used to support the findings of this study are included within the article.
